# Experiencing Microaggression: Invisibility, Distress, and Self-Stereotyping Among *Northeasterners* in India

**DOI:** 10.3389/fpsyg.2016.01995

**Published:** 2016-12-23

**Authors:** Khushbeen K. Sohi, Purnima Singh

**Affiliations:** Department of Humanities and Social Sciences, Indian Institute of Technology DelhiDelhi, India

**Keywords:** microaggression, invisibility, distress, self-stereotyping, *Northeasterners*

## Abstract

In the present times, the discrimination experiences of various marginalized groups tend to be characterized by subtle acts of disrespect and intolerance in addition to the traditional and more blatant incidents of violence. One such newer manifestation is microaggression. This research explored the impact of frequency of experiencing invisibility (i.e., feeling ignored or overlooked owing to one’s group membership) on distress among *Northeasterners* residing in Delhi. Further, the role of individual self-stereotyping as a moderator in the invisibility frequency-distress relationship was investigated. Moderation analysis suggested a significant moderating effect of individual self-stereotyping in the relationship between frequency of experiencing invisibility acts and the distress experienced by *Northeasterners*. In other words, experiencing invisibility caused distress for participants who saw themselves as prototypical of the *Northeasterners*. Interestingly, frequency of experiencing invisibility was associated with distress for all *Northeasterners*, however the size of this relationship was greater for *Northeasterners* who saw themselves as typical of their group.

## Introduction

Members of various social groups in various parts of the world as well as in India continue to encounter marginalization because of predetermined characteristics like caste, class, gender, religion, region, etc. Even in metropolitan cities, which are otherwise assumed to be multicultural egalitarian spaces, one witnesses such incidents. One such group is comprised of *Northeasterners* (or people from India’s Northeast region) residing in metropolitan cities like New Delhi who migrate in search for education/jobs. Instances of intolerance faced by this group in different parts of the country have found a wide coverage in the media. However, there is hardly any academic research that has attempted to explore the experiences of migration in this group, their confrontation with discrimination and the impact of such marginalization on them.

The present research is an attempt to address this gap by exploring incidents of microaggression (more specifically, invisibility, i.e., feeling ignored or overlooked owing to one’s group membership) in this group and the distress caused by such experiences. In addition to this, the research also aims at delineating the role of self-stereotyping as an in-group member in this process.

## Microaggression

In addition to overt and blatant acts of discrimination, psychologists are now also studying other more subtle expressions of discrimination. One such newer form which is the focus of the present work is microaggression- the brief and commonplace daily verbal, behavioral, and environmental indignities, whether intentional or unintentional, that communicate hostile, derogatory, or negative racial slights and insults to the target person or group (Sue et al., 2007). Chester M. Pierce coined the term microaggression in the 1970s to describe the commonplace subtle and often automatic “put-downs” and insults directed at the Black Americans. Although this term was then used specifically in the context of ‘racial’ microaggression, today microaggressions are studied in the context of various groups marginalized on the basis of gender, sexual orientation, physical disability, religion, or even class (Sue, 2010). Racial microaggressions can be understood as the everyday manifestation of racism on an interpersonal level, rather than the systemic racial oppression brought about thorough discriminatory laws and policies (Sue et al., 2007).

Acts of microaggression find different manifestations in the everyday life of marginalized groups. Sue et al. (2007) provided a microaggression taxonomy including acts such as assuming that the target person is of foreign origin (foreigner/not belonging), assuming that the target person is involved in criminal activities (criminality), ignoring the presence of or overlooking target group members (invisibility), assuming that the target person is low on intelligence (low-achieving/dysfunctional culture) etc. Torres-Harding et al. (2012) found these different forms to be related yet distinct. They suggested that instead of using a general racial microaggression factor, each of the factors be scored and examined independently. The present research focuses on the subtype of invisibility (i.e., feeling ignored or overlooked owing to one’s group membership). It also explores how individual self-stereotyping impacts the relationship between frequency of experiencing invisibility and distress caused by such events.

### Invisibility and Distress: Exploring the Linkages

Invisibility involves instances such as disregarding the presence of target group members in classrooms, dismissing their contributions and so on. The implicit message that is likely to get communicated through such acts is that they are insignificant and unnoticeable (Sue, 2010). These actions can become so routine and pervasive, that perpetrators may automatically respond in these ways in the presence of target group members. These acts serve as status reminders by implicitly conveying disregard and disrespect for the target group (Franklin and Boyd-Franklin, 2000). *Northeasterners* very often experience microaggression through invisibility which could result in experiences of marginalization. Sue (2010) notes that microaggressions involve the active manifestations of oppressive worldviews that perpetuate marginalization. Marginalization involves relegating a certain group of people to the periphery of social desirability and consciousness (Sue, 2010). It would not be an exaggeration to say that such groups remain ‘invisible’ from the mainstream, and thus, excluded from social, cultural and political spheres. These acts might seem trivial, but research has found these to have deleterious consequences for targets such as deterring the self-esteem of minority group members, producing anger and frustration, lowering subjective well-being, causing physical health problems and so on (Solorzano et al., 2000; Smedley and Smedley, 2005; Brondolo et al., 2008). With such marginalization comes a constant, continuous and cumulative experience of inferior status in various domains of life (Sue, 2010). These seemingly innocuous acts go on to act as daily hassles, which are known to have a harmful impact on individuals’ psycho-social functioning due to their persistent nature. Infact, research conducted on people of color has found that they experience subtle racism as more difficult to deal with than traditional racism (Dovidio and Gaertner, 1998; Salvatore and Shelton, 2007).

The stress caused by these acts of microaggression has been termed ‘microaggressive stress’ (Sue, 2010). The present research is specifically looking at the stress caused by frequency of experiencing invisibility. The impact of overt discrimination episodes on well-being of marginalized groups has been well explored and documented in psychology literature (Harrell et al., 2003; Greene et al., 2006; Okazaki, 2009). However, researchers are now also investigating the consequences of everyday race-related stressors (e.g., Sellers and Shelton, 2003; Ong et al., 2009). Marginal social status can cause the ‘invisibility syndrome’ owing to repeated encounters with instances of prejudice and discrimination (Franklin and Boyd-Franklin, 2000). Invisibility due to marginalization can have various negative consequences for target group members. Accumulated experiences of slights and indignities reinforce these feelings of being devalued owing to one’s group membership. Experiences of invisibility clearly indicate to minority community members that they are not recognized for their worth and their contributions and presence tend to be dismissed solely due to the group they belong to. It leaves a feeling of being lost in a crowd with one’s group identity being dismissed. As is known, recognition and approval are important social motives. So when these do not form a part of one’s experience and one’s group continues to be devalued, this could be an extremely stressful experience for group members. Being at the receiving end of microaggressions, especially not being recognized for one’s worth and being pushed to the periphery causes trouble to target group members as these reiterate their lower status in society and build a negative and hostile racial climate (Solorzano et al., 2000; Sue et al., 2007; Wang et al., 2011).

Torres-Harding and Turner (2014) noted that while a vast body of research has explored long-term links between racial microaggressions and health and well-being, fewer studies have examined the immediate stressful reactions or distress caused by experiences of microaggression. Microaggression theory suggests that more frequent microaggression experiences would result in more distress because of a presumed cumulative effect (Sue et al., 2007). So more frequently individuals experience slights, are overlooked and/or their contributions are dismissed owing to their group membership-all this can be labeled as invisibility- the greater is the likelihood of this to have a cumulative effect on distress experienced by individuals. While a lot of research on daily racial microaggressions has been conducted in the past decade (e.g., Kohli and Solórzano, 2012; Ong et al., 2013), empirical work assessing frequency of experiencing invisibility and its effect on distress among *Northeasterners* is still lacking. In an early study, Sohi and Singh (2015) studied microaggression experiences among *Northeasterners* and found experiences of microaggression to be negatively related to their social well-being. However, the specific effect of frequent experiences of invisibility on the distress caused has not been looked into. The present study thus, investigates the impact of frequency of experiencing invisibility on distress among *Northeasterners*.

## *Northeasterners* as a Marginalized Group

Men and women from India’s northeast migrate to cities like Delhi in large numbers for education and job pursuits. However, what has come to be known in the recent times is that *Northeasterners* are increasingly being made targets of intolerance, stereotyping and discrimination, both physical and verbal. In January 2014, Jamia Millia Islamia’s Centre for North East Studies and Policy Research and National Commission for Women jointly released a study titled *“Discrimination and Challenges before Women from North East India: Case Studies from four metros – New Delhi, Mumbai, Kolkata, and Bengaluru.”* Among other things, the study reported that nearly sixty percent of migrant *Northeastern* women have experienced some or the other form of harassment. Delhi, among the four metros studied, was reported as the most unsafe place by women. Eighty one percent of *Northeasterners* reported being victims of harassment in Delhi.

Based on his ethnographic study of Northeast migrants in Delhi, McDuie-Ra (2012) says that the experiences of *Northeasterners* differ from the discrimination experiences of other migrant minorities in India. What contributes to this difference according to him is the notion of ‘race.’ He goes on to state that *Northeasterners* are very often perceived to be racially different from other inhabitants of the land. They tend to be perceived as the ‘outsider’ within the Indian heartland. Their distinct physical features and cosmopolitan lifestyle fuel this misinformed perception. The current study borrows McDuie’s conceptualization of the discrimination faced by *Northeasterners* as being race-related to explore the question under study.

## The Role of Self-Stereotyping

Group identification can be understood as the extent to which an individual attaches value and importance to a group membership (Tajfel, 1978; Turner et al., 1987). Instead of studying group identification as a unidimensional construct, researchers are now explicating several different components of the construct. One such influential model has been provided by Leach et al. (2008). They presented a five-component model of identification which includes (a) individual self-stereotyping, or how similar one perceives oneself to be to the average or prototypical members of the in-group; (b) in-group homogeneity, or how much one perceives the group as sharing commonalities that make the group a homogeneous entity; (c) satisfaction, or how positively one feels about the in-group and one’s membership in it; (d) solidarity, or one’s psychological bond with and commitment to the other members in the group; and (e) centrality, or the salience and importance of one’s in-group membership to one’s self-concept. Of Leach et al.’s (2008) five components of identification, the present research focuses on the component of individual self-stereotyping. Identification with a group involves both, a self-categorization process that includes the individual in the group as well as the individual’s perception of oneself in terms of that group membership (Leach et al., 2008). According to the tenets of the self-categorization theory (Turner et al., 1987), “in-group identification is indicated by a “depersonalized” self-perception, whereby individuals come to “self-stereotype” themselves as similar to other members of their in-group” (Leach et al., 2008). Thus, individual self-stereotyping or perceiving oneself as similar to and having things in common with the prototypical group member is an important component of identification with a group. Leach et al. (2008) further state that the depersonalization and psychological inclusion in the in-group brought about by stereotyping oneself as a typical group member is likely to bring about a sense of common fate with the rest of the group. This in turn means that this form of self-definition would lead individuals to get emotionally involved with the in-group’s successes and failures.

With respect to the role of identification with the in-group for individuals who experience group-based discrimination, two different perspectives have emerged. The first of these draws on in-group identification research which suggests that strong group identity may be a buffer against potential negative consequences of discrimination. It follows that self-stereotyping as an in-group member may serve as a buffer against potential negative consequences of discrimination. The rejection-identification model (RIM; Branscombe et al., 1999) asserts that perceiving pervasive discrimination against the in-group may enhance identification with one’s group which may be seen as self-stereotyping as an in-group member. This may satisfy people’s need for belonging and inclusion and as a result foster psychological well-being (Branscombe et al., 1999). Embracing a relevant group identity through self-stereotyping then serves an adaptive purpose that helps disadvantaged group members thwart the harmful impact of perceived discrimination.

According to the second perspective, strongly identifying with one’s group through for instance self-stereotyping oneself as an in-group member is likely to exacerbate distress owing to acts of discrimination. Supporting the argument that individuals with a strong group identity experience greater distress due to discrimination as compared to individuals who are not strongly identified with the in-group, McCoy and Major (2003) found that perceiving prejudice against the in-group negatively impacted the self-evaluative emotions (depression, self-esteem) of women who were highly identified with the in-group. This effect was not found among women low in gender identification. This essentially testifies that for individuals with a strong sense of group identification, any threat to the group is perceived as a threat to the self.

## Hypotheses

(1) Frequency of experiencing invisibility (i.e., feeling ignored or overlooked owing to one’s group membership) will be positively related to distress.
(2) Self-stereotyping as an in-group member will moderate the effect of frequency of experiencing invisibility on distress.

## Materials and Methods

### Participants and Procedure

Two hundred and twenty-four (121 females; 103 males) *Northeasterners* in the age group of 19–31 years who had been residing in Delhi for a minimum of 1 year at the time of the study took part in the research. They were graduates, post-graduates and employed. Participants were approached through referrals given by participants and requested to participate in the study. They were briefed that the study was being carried out to explore the experiences of *Northeasterners* in Delhi. Participation in the study was voluntary. Informed consent was obtained from the participants prior to the study.

### Measures

#### Frequency of Experiencing Invisibility

The present study used the Racial Microaggressions Scale (RMAS; Torres-Harding et al., 2012) to assess frequency of experiencing invisibility or how often participants had experiences of invisibility. Invisibility refers to experiences where individuals feel they were overlooked, invalidated, or their views and contributions were dismissed because of their group membership. This contributes to feeling marginalized, delegitimized and devalued by others. We reworded the eight items of the invisibility subscale to make them relevant to the sample of the present study. This subscale includes items such as *“We are ignored in college/university or work environments because of our regional background,” “Our contributions are dismissed or devalued because of our regional background.”* To assess the frequency of experiencing invisibility, for each item subjects indicated how often they had experienced the stated event (never; rarely; sometimes; often). The reliability index (Cronbach’s α) for invisibility frequency in this study was found to be 0.81. CFA results were also acceptable (GFI = 0.95; CFI = 0.94; RMSEA = 0.08; χ^2^/d.f. = 2.46)^1^.

#### Individual Self-Stereotyping

The study used two items measuring individual self-stereotyping from Leach et al.’s (2008) five-component scale. On a scale ranging from 1 (strongly dis*agree*) to 4 (*strongly agree*), participants indicated their agreement with the items *“I have a lot in common with the average Northeastern person*” and *“I am similar to the average Northeastern person.”* The reliability index (Cronbach’s α) for individual self-stereotyping in this study was found to be 0.75.

#### Distress

To assess the distress caused due to frequency of experiencing invisibility, the present study used the RMAS (Torres-Harding et al., 2012). We included eight items, for e.g., *“We are ignored in college/university or work environments because of our regional background,” “Our contributions are dismissed or devalued because of our regional background.”* Participants were required to indicate how stressful, upsetting or bothersome the experiences of invisibility were (not at all; a little; moderate level; high level). The distress subscales tap into stated stressfulness or the self-reported stressfulness of microaggression events (Torres-Harding and Turner, 2014). The reliability index (Cronbach’s α) for distress in this study was found to be 0.82. CFA results were also acceptable (GFI = 0.93; CFI = 0.91; RMSEA = 0.09; χ^2^/d.f. = 3.09).

## Results

This study examined the moderating role of self-stereotyping in the relationship between frequency of experiencing invisibility and distress. **Table 1** features means, standard deviations, and correlations among all variables. As can be inferred from **Table 1**, *Northeasterners* in the present study reported high frequency of experiencing invisibility (*M* = 1.74) and distress (*M* = 1.78). Both these scales were 4-point scales ranging from 0–3. Hence, any score above the mid-point (i.e., 1.5) has been considered a high score. They also reported high levels of individual self-stereotyping (*M* = 2.89). This was again a 4-point scales ranging from 1–4. Hence, any score above the mid-point (i.e., 2) has been considered a high score.

**Table 1:** Means, standard deviations, and correlations.

	Scale	*M*	*SD*	1	2	3
1. Frequency of Experiencing Invisibility	0–3	1.74	0.63	–	0.11	0.83^∗∗^
2. Individual Self-Stereotyping	1–4	2.89	0.59		–	0.07
3. Distress	0–3	1.78	0.72			–

*Columns labeled 1, 2, and 3 show correlation between variables. The numbers 1, 2, and 3 refer to the variables frequency of experiencing invisibility, individual self-stereotyping, and distress, respectively. ^∗∗^p < 0.01.*

In line with hypothesis 1, we found a significant positive correlation between frequency of experiencing invisibility and distress.

We tested hypothesis 2 using a moderation analysis, performed with the help of the SPSS macro PROCESS (model 1) developed by Hayes (2012). The predictors were mean-centered for this analysis. It was hypothesized that self-stereotyping as an in-group member would moderate the consequences of frequency of experiencing invisibility. This hypothesis was supported. It was also found that as self-stereotyping increased, frequency of invisibility experiences had a more positive impact on distress.

There was a significant main effect of frequency of experiencing invisibility on distress, *b* = 0.94, CI [0.87 – 1.02]. But there was no significant main effect of individual self-stereotyping on distress, *b* = -0.03, CI [-0.13 – 0.06]. The interaction between frequency of experiencing invisibility and individual self-stereotyping had a significant effect on distress, *b* = 0.16, CI [0.01 – 0.32]. To probe this interaction effect further, PROCESS was used to estimate the effect of frequency of experiencing invisibility on distress at the mean of the moderator, and at one standard deviation below the mean and one standard deviation above the mean. As can be seen in **Table 2** below, increased frequency of experiencing invisibility increased distress among participants who reported high individual self-stereotyping.

**Table 2:** Interaction effect and conditional effect.

	Coefficient	*SE*	*t*	*p*	LLCI	ULCI
Interaction (Frequency of experiencing invisibility × Individual self-stereotyping)	0.16	0.08	2.08	0.04	0.01	0.32

**Individual Self-Stereotyping**	**Effect**	***SE***	***t***	***p***	**LLCI**	**ULCI**

0.5933	0.8454	0.0682	12.4006	0.0000	0.7110	0.9797
0.0000	0.9414	0.0374	25.1988	0.0000	0.8678	1.0150
0.5933	1.0374	0.0492	21.0875	0.0000	0.9405	1.1344

*p = 0.0000 stands for p < 0.0001.*

It was also observed that increased frequency of experiencing invisibility increased distress among participants who reported low and moderate levels of individual self-stereotyping as well. Simple slopes showed that the association between frequency of experiencing invisibility and distress was positive when individual self-stereotyping was 1 SD above the mean, *B* = 1.04, CI [0.94 – 1.13], positive at the mean, *B* = 0.94, CI [0.87 – 1.02] and positive also at 1 SD below the mean, *B* = 0.84, CI [0.71 – 0.98].

**FIGURE 1:**
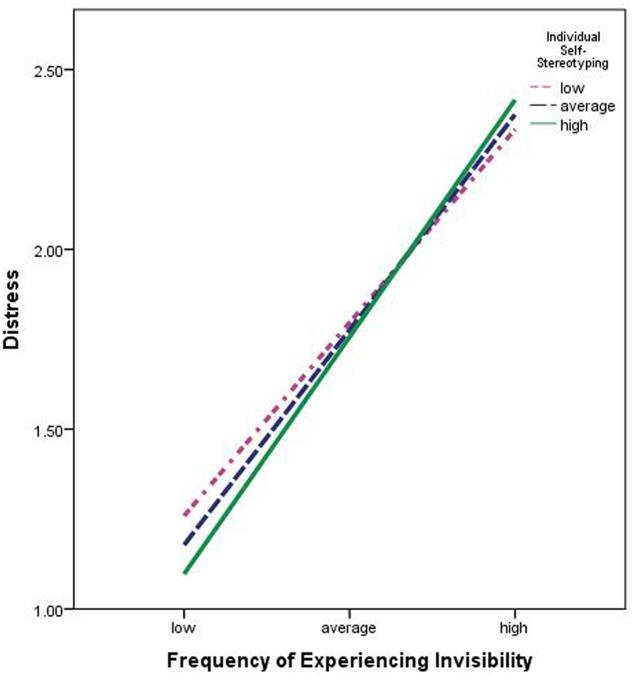
**The moderating effects of individual self-stereotyping on the association between frequency of experiencing invisibility and distress**.

In other words, it can be said that the positive relationship between invisibility and distress would get stronger in the case of participants who are higher on self-stereotyping themselves as *Northeasterners* as compared to the participants who self-stereotype themselves to a lesser degree. However, frequency of experiencing invisibility would nevertheless always be associated with distress. Only the size of this relationship would vary as a consequence of the moderator, individual self-stereotyping. This also becomes evident from the **Figure 1**. The slope linking frequency of experiencing invisibility to distress is steeper among those higher on self-stereotyping. That is, the effect of experiencing invisibility appears to be larger among those relatively higher on self-stereotyping than among those relatively lower on self-stereotyping.

## Discussion

The present paper aimed to investigate the experiences of invisibility encountered by *Northeasterners* in Delhi. To this end, we explored the role of individual self-stereotyping in moderating the effect of frequency of experiencing invisibility on distress. *Northeasterners* are a marginalized group in the social fabric of Delhi, the place where the study was conducted. Their distinctive appearance and different lifestyle not only lead them to be viewed as the other or the outsider, but this otherness invokes and blends in with the ill-informed opinions about the northeast that many a people hold in the heartland (McDuie-Ra, 2012). In the present study, we found a positive relationship between frequency of experiencing invisibility incidents and distress. Torres-Harding and Turner (2014) in a study of Latinos, African Americans, Asian American, and multiracial populations found similar results wherein higher invisibility frequency was associated with higher distress. People seek recognition and social approval. So when these are denied, it is bound to be a stressful experience. Feelings of being excluded from various social realms tend to foster alienation, conflict and decreased well-being.

Further, it was found that self-stereotyping as an in-group member moderated the impact of frequency of experiencing invisibility on distress such that, more frequent experiences of invisibility caused distress for participants who were high on self-stereotyping themselves as *Northeasterners*. It is well documented in social psychology research that we tend to be more attuned to environmental cues that are relevant to an important aspect of our identity (self-categorization theory, Turner et al., 1987). Instances of racial microaggression might serve as one such cue. Research on various minority groups has found a similar effect where highly group identified individuals have been found to be more attuned to both overt and subtle acts of discrimination (Operario and Fiske, 2001; Sellers and Shelton, 2003; Sellers et al., 2003) and respond more negatively to such events.

However, it was also found that the effect of experiencing invisibility on distress appeared to be larger among those relatively higher on self-stereotyping than among those relatively lower on self-stereotyping. So although self-stereotyping did moderate the relationship between frequency of experiencing invisibility and distress, this effect was also found at relatively low to high levels of self-stereotyping. There can be several possible reasons for this. Even when *Northeasterners* do not strongly stereotype themselves as members of this category, acts of discrimination directed toward them indicate that they are being seen by others around them as representative of the category ‘*northeasterner.*’ Constantly being made the target of subtle biases and acts of discrimination owing to one’s group membership, is bound to have an adverse effect on one’s daily adjustment and over all functioning in society. It becomes a daily hassle dealing with which puts a strain on one’s psychological and emotional resources and well-being.

The findings of the study point toward some important implications. Considering the negative effect that microaggression experiences can exert, it is extremely important firstly, that locals be educated about which acts contribute to microaggression. Secondly, awareness needs to be raised about the harm that such acts can cause to members of minority groups. It has been observed that as a consequence of being ignored and devalued, minorities all over the world are zealously adopting distinct markers of identity in order to re-assert their identity. In other words, they want to be accepted as member of their respective groups. It is this recognition and acknowledgment that can contribute to establishing harmonious relations between all groups in a society.

This research holds immense relevance for the following reasons. It addressed an important gap in literature by investigating the impact of frequency of experiencing invisibility among *Northeasterners* in India. With the frequent acts of intolerance toward this group, the importance of such a research only gets reiterated. In addition, the present study attempted to address a newer form of discrimination concerning microaggression (more specifically, the invisibility dimension of microaggression). It is important to expand research efforts in this area as it is these subtle biases and stereotypes that precede or set the stage for more gruesome acts of violence against members of this group (Sohi and Singh, 2015). As more and more research provides evidence that clandestine racial discrimination is perceived as more harmful than open racism (Major et al., 2003), the importance of work focused on microaggressions and its various dimensions can hardly be undermined. Also, research looking explicitly at the consequences of experiencing invisibility (or ignorance from the mainstream) is still very limited. This study thus, adds to our understanding of this important dimension of microaggression. In addition to this, the findings of this study support earlier work which has established microaggression to be a stressor for target group members. However, this study goes one step ahead and shows that identification with the in-group is an important factor here. This aspect has not been sufficiently explored by previous work in this domain. The study also focused on the immediate distress caused by such events rather than on long-term consequences, an area which again has received only limited research attention until now. It is time we became proactive about ensuring inclusive spaces of education, work and leisure for *Northeasterners* as well as other marginalized groups in our society.

## Ethics Statement

Approval from ethics committee could not be solicited for the present work as our institute does not provide this facility for doctoral students in the department of Humanities and Social Sciences. Informed consent was obtained from all study participants prior to conducting this research. Also, it was reiterated to them that participation in the study was absolutely voluntary and that they could discontinue participation at any time.

## Author Contributions

All authors listed, have made substantial, direct and intellectual contribution to the work, and approved it for publication.

## Conflict of Interest Statement

The authors declare that the research was conducted in the absence of any commercial or financial relationships that could be construed as a potential conflict of interest.

## Footnotes

1 CFA, confirmatory factor analysis; GFI, goodness-of-fit index; CFI, comparative fit index; RMSEA, root mean square error of approximation.
